# Surveillance of Pathogenicity of *Rhizoctonia solani* Japanese Isolates with Varied Anastomosis Groups and Subgroups on *Arabidopsis thaliana*

**DOI:** 10.3390/life12010076

**Published:** 2022-01-06

**Authors:** Mai Mohsen Ahmed Abdelghany, Maria Kurikawa, Megumi Watanabe, Hidenori Matsui, Mikihiro Yamamoto, Yuki Ichinose, Kazuhiro Toyoda, Yusuke Kouzai, Yoshiteru Noutoshi

**Affiliations:** 1 Graduate School of Environmental and Life Science, Okayama University, Okayama 700-8530, Japan; pazi0ct5@s.okayama-u.ac.jp (M.M.A.A.); po7l1am9@okayama-u.ac.jp (M.W.); hmatsui@okayama-u.ac.jp (H.M.); myama@okayama-u.ac.jp (M.Y.); yuki@okayama-u.ac.jp (Y.I.); pisatin@okayama-u.ac.jp (K.T.); yusuke.k@affrc.go.jp (Y.K.); 2 National Institute of Oceanography and Fisheries (NIOF), Alexandria 21556, Egypt; 3 Department of Agriculture, Okayama University, Okayama 700-8530, Japan; p3io9bg4@s.okayama-u.ac.jp

**Keywords:** *Rhizoctonia solani*, anastomosis group, phytohormones, pathogenicity, *Arabidopsis thaliana*

## Abstract

*Rhizoctonia solani* is a necrotrophic plant pathogen with a wide host range. *R. solani* is a species complex consisting of thirteen anastomosis groups (AGs) defined by compatibility of hyphal fusion reaction and subgroups based on cultural morphology. The relationship between such classifications and host specificity remains elusive. Here, we investigated the pathogenicity of seventeen *R. solani* isolates (AG-1 to 7) in Japan towards *Arabidopsis thaliana* using leaf and soil inoculations. The tested AGs, except AG-3 and AG-6, induced symptoms in both methods with variations in pathogenicity. The virulence levels differed even within the same AG and subgroup. Some isolates showed tissue-specific infection behavior. Thus, the AGs and their subgroups are suggested to be not enough to define the virulence (host and tissue specificity) of *R. solani*. We also evaluated the virulence of the isolates on Arabidopsis plants pretreated with salicylic acid, jasmonic acid, and ethylene. No obvious effects were detected on the symptom formation by the virulence isolates, but ethylene and salicylic acid slightly enhanced the susceptibility to the weak and nonvirulent isolates. *R. solani* seems to be able to overcome the induced defense by these phytohormones in the infection to Arabidopsis.

## 1. Introduction

*Rhizoctonia solani* Khün is a necrotrophic destructive plant pathogen with a nearly unlimited host range worldwide [1]. It is a soil-borne fungus causing different symptoms such as seedlings damping-off; root, crown, and stem rots; in addition to foliar and sheath blights [2]. This pathogen can survive in soil as sclerotia or mycelia within diseased plant material during unfavorable environmental conditions for several years. It does not produce asexual spores and rarely forms sexual spores in nature. *R. solani* is a species complex composed of many related but genetically isolated groups [3,4]. These groups are known as anastomosis groups (AGs). The hyphae of the same AG can fuse or anastomose with each other to expand their genetic diversity [5,6]. To date, *R. solani* is divided into 13 AGs designated as AG-1 through 13, as the AG-BI group has been integrated into AG-2 [7]. The single anastomosis group can be divided into multiple subgroups according to their pathogenicity, host specificity, cultural morphology, nutritional requirements, optimum temperature, and hyphal anastomosis frequency [8,9].

*R. solani* isolated from rice sheath blight belongs to AG-1 IA, but it also has been reported as pathogens of soybean and some *Brassica* species [10,11]. From Brassicaceae, AG-2-1 isolates were frequently isolated [8,12,13]; however, this fact does not necessarily prescribe the host range or preferential host species of a particular AG and subgroup. In fact, AG-2-1 was also found to infect pea, wheat, and barley [14,15]. To clarify if there is a relationship between host specificity and AGs and subgroups, it is needed to survey the infectivity of *R. solani* isolates to different plant species. The pathogenicity of 35 *R. solani* isolates, belonging to AG-1, AG-2, AG-3, and AG-4, was tested on kidney beans, soybeans, red pepper, radish, sugar beet, and cabbage, and the virulence of the same isolate was varied on the different hosts [16]. Keijer et al. surveyed the host range of 32 *R. solani* isolates (ranging from AG-1 to 6) using two Brassicaceae (cauliflower and *A. thaliana*) and three Solanaceae (eggplant, tomato, and potato) plant species, and detected a certain degree of pathogenicity to plant species in the same family as the originally isolated plant species [17]. *R. solani* isolates belonging to AG-8 and AG-2-1 isolated from wheat, barley, lentil, fallow, onion, canola, and chickpea from Washington State in the US were evaluated for their infectivity to wheat and canola [18]. All the AG-2-1 isolates, but not the AG-8, except a single isolate, showed symptoms in canola. Moreover, AG-2-2 IIIB isolated from maize was found to be very aggressive against cauliflower [19]. In the same context, Kidd et al. and Foley et al. demonstrated that AG-2-1 but not AG-8 is pathogenic to *A. thaliana* in soil inoculation [20,21]. On the other hand, Kumar et al. showed that *A. thaliana* was susceptible to AG-2-1 and AG-8 C1 when it was inoculated to soil [22]. AG-8 that causes bare patch in cereals and legumes crops was found to be very aggressive to canola [23]. We previously tested whether the *R. solani* Japanese isolates infect leaves of *Brachypodium distachyon*, a monocotyledonous model plant [24]. The AG-1 IA isolates identified from rice and sudangrass, AG-2-3 from wheat, AG-4 IIIA from cauliflower, and AG-5, AG-6, and AG-2 BI from the soil showed virulence towards *B. distachyon* leaves with varied pathogenicity levels. The host preference of particular AGs appears to be detected at a certain level; however, the relationship between the pathogenicity of *R. solani* and AG and subgroup remains elusive.

In addition to host specificity, tissue specificity can also provide important aspects for the virulence mechanism of *R. solani*. The *R. solani* AG-2-1 was found to be virulent to Arabidopsis detached leaves and induced severe necrotrophic symptoms, while its colonization was restricted in roots when it was inoculated into the soil [20]. The relationship between *R. solani* AGs and subgroups along with tissue-specific virulence is also unclear.

In the plant immunity responses, defense-associated phytohormones such as salicylic acid (SA), jasmonic acid (JA), and ethylene (ET) play important roles [25,26]. The SA-induced defense pathway is mainly effective against biotrophic pathogens, whereas the JA/ET-induced defenses are typically most effective against necrotrophic pathogens that kill plant cells to obtain nutrients [27,28,29,30]. The investigation of required plant defense mechanisms in plants to particular pathogens is helpful to understand a pathogen’s infection strategy. In *A. thaliana*, the symptoms induced by *R. solani* AG-2-1 did not alter in the mutants related to auxin, camalexin, abscisic acid, SA, and ET/JA pathways [20]. This suggests that *R. solani* AG-2-1 could successfully suppress defense responses and the infection was established at the maximum level that makes it impossible to detect the contribution of a particular defense signaling cascade by this method. On the other hand, in these mutants, the susceptibility to *R. solani* AG-8 was not induced. However, further genetic analyses using triple mutants revealed that ET, JA, and *PENETRATION2*-mediated defense pathways are the foundation of Arabidopsis nonhost resistance to *R. solani* AG-8 [20]. Similarly, the contribution of PEN1, PEN2, and PEN3 to nonhost resistance in *A. thaliana* leaf to *R. solani* AG-1 IA has been demonstrated [31]. The loss of DORN1/P2K1 or RBOH proteins enhanced the susceptibility of *A. thaliana* roots to soil-inoculated AG-8 [22]. We previously found that foliar application of SA to *B. distachyon* and rice could induce sheath blight resistance caused by *R. solani* AG-1 IA [24]. Consistently, transgenic rice plants of *NahG*, bacterial SA hydroxylase gene, showed decreased resistance level to the pathogen. This suggests the possible biotrophic interaction in this inoculation system; however, it remains unknown if *R. solani* isolates behave similarly during infection on dicotyledonous plants such as *A. thaliana.*

In this study, we surveyed the potential pathogenicity of seventeen Japanese *R. solani* isolates with varied AGs and subgroups collected from various diseased plants and soils on Arabidopsis. The virulence towards both leaves and roots was tested. In addition to *R. solani* AG-2, which is frequently isolated from Brassicaceae, AG-1, AG-4, AG-5, and AG-7 were also found to be pathogenic to Arabidopsis. The isolates with the same AGs and subgroups represented different pathogenicity. Moreover, most of the isolates showed different pathogenicity towards leaves and roots. We also investigated the induced resistance and susceptibility by phytohormones (SA, JA, and ET) to the *R. solani* isolates. The pretreatment of all the phytohormones did not induce resistance to the virulent isolates in Arabidopsis. Meanwhile, the treatment with ET slightly induced susceptibility to the weak and nonvirulent isolates.

## 2. Materials and Methods

### 2.1. Fungal Isolates and Plant Materials

Seventeen *Rhizoctonia solani* isolates were obtained from the Genebank of the National Agricultural Research Organization (NARO) in Japan. The sources of these isolates are summarized in Table 1. 

*R. solani* isolates were maintained on potato dextrose agar (PDA) medium (24 g/L Difco™ potato dextrose broth and 2% agar). Arabidopsis seeds were surface-sterilized using sodium hypochlorite for five minutes, then suspended in sterile water and washed five times. The seeds were stored for three days at 4 °C in dark condition before sowing. Arabidopsis seeds were plated on half-strength Murashige and Skoog (1/2MS) medium plates (2.23 g/L MS salt (Nihon Pharmaceutical, Tokyo, Japan) enriched with 0.1% (*v*/*v*) Gamborg’s vitamin solution (Sigma-Aldrich, St. Louis, MO, USA), 1% (*w*/*v*) sucrose, 0.05% MES, and 0.8% agar, pH 5.7 with KOH). Two-week-old Arabidopsis seedlings were transplanted into the soil (Supermix-A; Sakata Seed, Kanagawa, Japan) and grown for 2–3 weeks in a long-day growth chamber with LED lights (Nippon Medical & Chemical Instruments, Osaka, Japan) under a 16 h light/8 h dark photoperiod at 23 °C.

### 2.2. Inoculation Tests

Leaf inoculation assay was performed by using detached rosette leaves prepared from 4-week-old Arabidopsis plantlets. The leaves were placed onto moistened Whatman filter paper in a petri dish, and the cut edges were wrapped with a moist paper wiper (KimWipes; Nippon Paper Crecia, Tokyo, Japan). Mycelial plugs (6 mm diameter) were made with a biopsy punch (BP-30F; Kai Corporation, Tokyo, Japan) from *R. solani* grown on PDA medium when the mycelial growth reached the plate edges, and they were placed on the middle of the detached Arabidopsis leaf [32]. The plates were covered and placed in a tray with a clear plastic lid lined with moist paper towels to keep high humidity. Plates were incubated at 23 °C in a long-day growth chamber. The developed symptoms were observed and photographed daily until the fifth day post-inoculation (dpi). Noncolonized PDA plugs were used as a control. The assay was performed at least three times with three biological replicates each to confirm the reproducibility. 

Soil inoculation assay was carried out following the method of Bowyer et al. [33]. Briefly, 70 mL capacity pots were filled with soil, and four mycelial PDA plugs (6 mm diameter) were inoculated. The pots were kept in a container with a clear plastic lid at 23 °C for 6 days. Two-week-old *A. thaliana* seedlings grown on MS agar were transplanted into the inoculated soil. We planted two plants per one pot. Photographs were taken at 0, 3, 5, 7, 10, and 14 dpi (only images at 0, 7, and 14 dpi are shown in the results). Soil containing four noncolonized PDA plugs was used as a control. The assay was carried out three times with three biological replicates each to confirm the reproducibility of the results.

### 2.3. Phytohormones Treatments

Sodium salicylate (Wako, Osaka, Japan), methyl jasmonate (Wako), and ethephon (Sigma-Aldrich), an ethylene generator, were used. The phytohormones were dissolved in dimethyl sulfoxide (DMSO), then diluted with distilled water to prepare 1 mM solutions (final DMSO concentration is 0.1%), which were supplemented with 0.04% (*v*/*v*) tween-20. Four-week-old *A. thaliana* plants grown on soil were sprayed with the phytohormone solutions and incubated for 48 h at the same growth condition. Then, the detached rosette leaves were prepared and inoculated with *R. solani* PDA plugs as described above [24]. For the soil inoculation method, 10-day-old *A. thaliana* seedlings grown on 1/2MS were sprayed with the phytohormones solutions and kept in the same growth condition for 48 h. Then, seedlings were transplanted in the *R. solani*-inoculated soil as described before.

## 3. Results

### 3.1. Pathogenic Behavior of the Rhizoctonia solani Isolates on Arabidopsis Leaves

To evaluate the relationship between host specificity and AGs and subgroups, we collected Japanese isolates of *R. solani* belonging to seven different AGs (AG-1 to 7) from NARO Genebank: four AG-1 isolates, eight AG-2 isolates, and single isolates for each of AG-3, AG-4, AG-5, AG-6, and AG-7 that were isolated from diseased crops and soil (Table 1). In this study, we firstly evaluated their infectivity to leaves of *A. thaliana* Col-0. 

**Figure 1:**
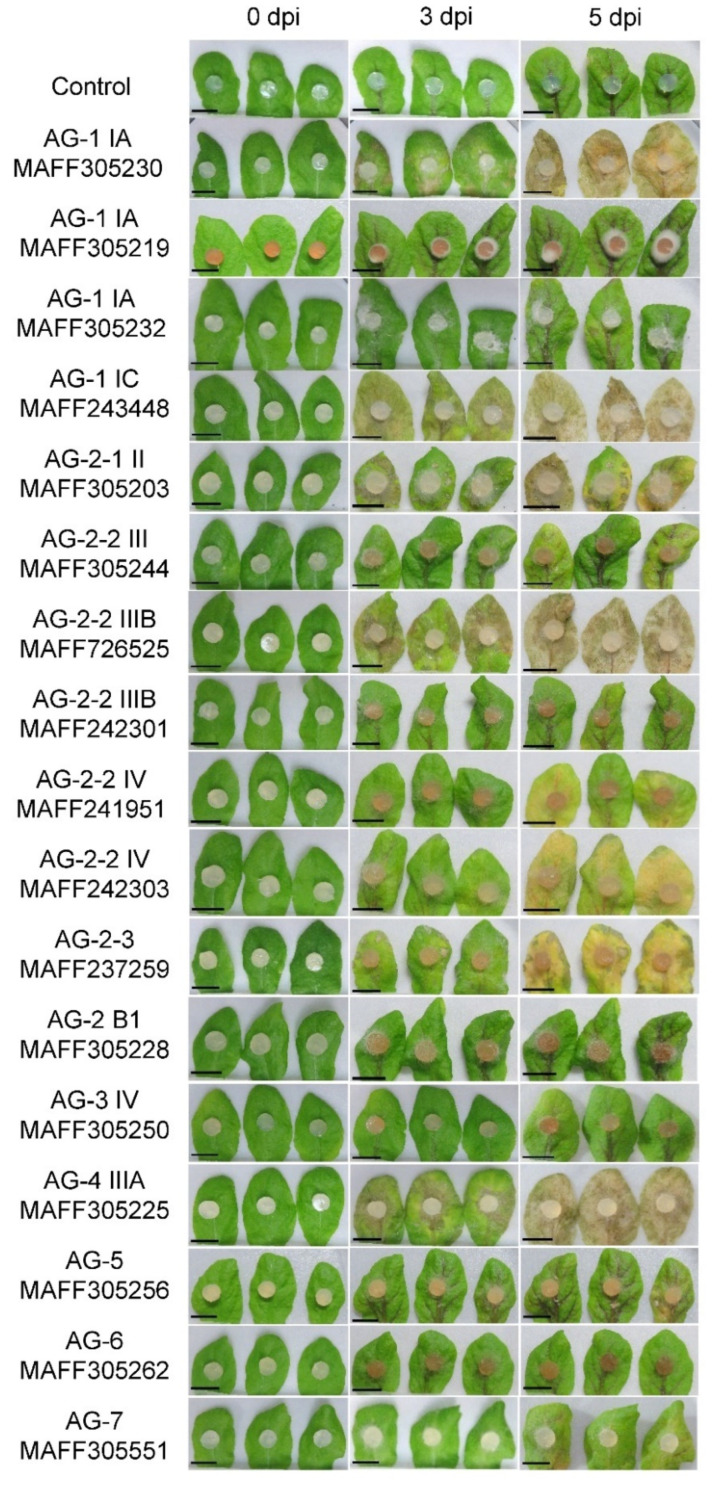
Infectivity of *Rhizoctonia solani* Japanese isolates in detached leaves of 4-week-old *Arabidopsis thaliana*. Leaves were inoculated with 6 mm mycelial PDA plugs and incubated in a humid condition at 23 °C for 5 days. Photographs were taken at 0, 3, and 5 days post-inoculation (dpi). Plain PDA plugs without fungus were used as a control. The assays were performed three times with three biological replicates. Bars, 1 cm.

The mycelial plugs of these isolates were inoculated to Arabidopsis detached leaves as described in the method section, and the photographs at 0, 3, and 5 dpi are shown in Figure 1. We classified the infection aggressiveness of each isolate into four levels, highly aggressive (+++), moderately aggressive (++), weak aggressive (+), and nonaggressive (−) as described in Table 1. We considered that the strength of the detected virulence in our assay possibly depends on the growth speed of each isolate. To confirm this point, we measured the growth speed of the mycelium of each *R. solani* isolate (Table 1). As a result, it seems not to be correlated with their virulence.

The infection symptoms were observed in the *R. solani* isolates AG-1, AG-2, AG-4, AG-5, and AG-7 but not AG-3 and AG-6. Note that the virulence levels of AG-5 and AG-7 were categorized as weak aggressive, but actually, they were very weak towards Arabidopsis leaves until the fifth dpi. Therefore, AG-1, AG-2, and AG-4 showed obvious symptoms in Arabidopsis leaves. Interestingly, the virulence level differed within the same AGs and subgroups. In this study, we used three AG-1 IA isolates (two AG-1 IA isolates from rice (MAFF305230 and 305219) and one AG-1 IA isolate from sudangrass (MAFF305232)) and, among them, only a single isolate (MAFF305230) exhibited severe necrotic lesion. As for AG-2-2 IIIB, three different isolates, MAFF305244 from rice, MAFF726525 from Broccoli, and MAFF242301 from welch onion, were tested. In this case, only a single isolate, MAFF726525, was found to be highly pathogenic. These results indicate that the AGs and subgroups are unrelated to the virulence of *R. solani* to a particular host.

### 3.2. Pathogenic Behavior of the Rhizoctonia solani Isolates on Arabidopsis Roots

**Figure 2:**
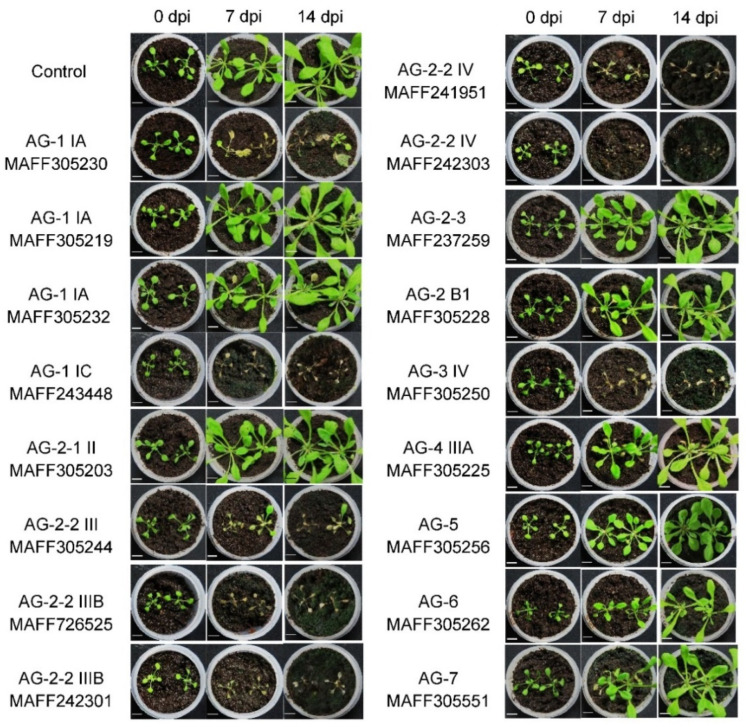
Infectivity of soil inoculated with *Rhizoctonia solani* Japanese isolates in young seedlings of *Arabidopsis thaliana*. PDA plugs prepared from *R. solani* grown in medium were inoculated to soil and incubated for 6 days. Then, 2-week-old Arabidopsis seedlings grown on MS agar plate medium were transplanted. Photographs were taken at 0, 7, and 14 days post-inoculation (dpi). Plain PDA plugs without fungus were used in the control treatment. The assays were performed three times with three biological replicates.

Next, we inoculated *R. solani* isolates into the soil prior to transplanting Arabidopsis seedlings to evaluate their pathogenicity toward underground tissues. The photographs of the pots were taken at 0, 7, and 14 dpi as shown in Figure 2. The infection aggressiveness of the isolates in this assay was categorized into three levels, highly aggressive (+++), moderately aggressive (++), and nonaggressive (−) as summarized in Table 1. The *R. solani* isolates AG-1, AG-2, and AG-4 were strongly virulent in this infection system. On the other hand, AG-3, AG-5, AG-6, and AG-7 did not exhibit obvious symptoms. The AG-5 and AG-7 represented weak virulence on leaf tissue, but their pathogenicity was not detected in the root inoculation assay, although we cannot exclude the possibility of their weak virulence to root tissue, which can be detected at a microscopic level. The AG-1 IA (MAFF305230), AG-1 IC (MAFF243448), AG-2-2 IIIB (MAFF726525), AG-2-2 IV (MAFF241951 and MAFF242303), and AG-4 IIIA (MAFF305225), which were virulent in leaf inoculation, were also highly pathogenic to underground plant parts. The AG-1 IA isolates (MAFF305219 and MAFF305232), which are nonpathogenic isolates on leaves, also did not induce symptoms in the soil inoculation assay. The AG-2-1 II (MAFF305203) and AG-2-3 (MAFF237259) were pathogenic to leaves but nonpathogenic to roots, while the AG-2-2 IIIB (MAFF305244 and MAFF242301) were nonpathogenic to leaves but pathogenic to roots. According to the results in the leaf inoculation assay, there is no correlation between virulence and AGs and their subgroups. Comparing the results obtained from the two inoculation methods, the virulence of particular *R. solani* isolates would be varied towards different plant tissues.

### 3.3. The Effects of Exogenously Applied SA, JA, and ET on the Resistance to Rhizoctonia solani in Arabidopsis Leaves

**Figure 3:**
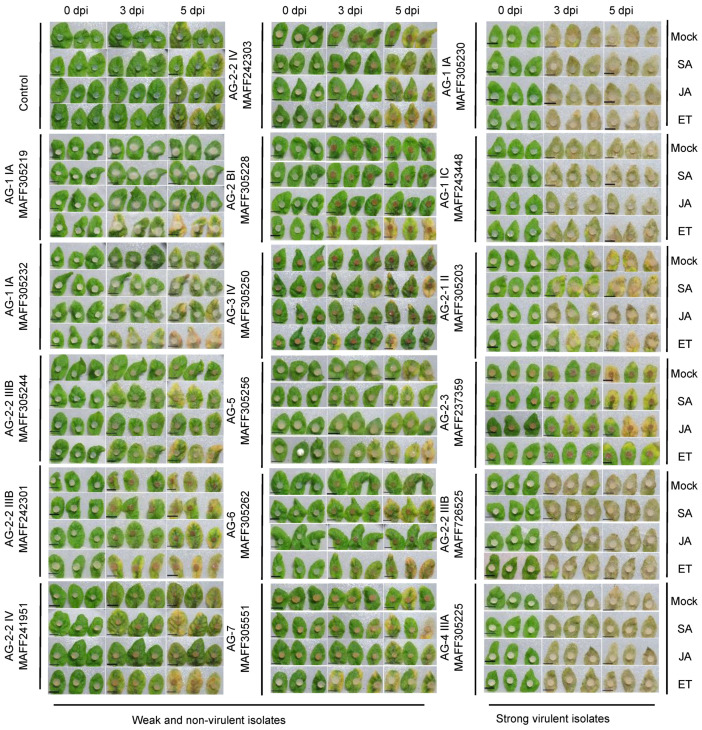
Effects of pretreatment of the defense-related phytohormones, salicylic acid (SA), jasmonic acid (JA), and ethylene (ET), on disease resistance of *Arabidopsis thaliana* leaves against *Rhizoctonia solani* Japanese isolates. Four-week-old Arabidopsis plants were sprayed with SA, JA, or ET (1 mM each; final concentration of DMSO was 0.1%). After 48 h, the rosette leaves were detached and put in Petri dishes. Then, mycelium plugs prepared from *R. solani* grown in PDA medium were inoculated. The left and middle columns were weak or nonvirulent isolates, and the right column was strong virulent isolates, determined by the assay in Figure 1. Photographs were taken at 0, 3, and 5 days post-inoculation (dpi). Plain PDA plugs without fungus were used as a control. The assay was replicated three times.

Gene knockout approach is useful to understand the virulence mechanism of *R. solani* at a molecular level, but currently, it is impossible due to its multinucleate nature and the unavailability of an efficient transformation method. In an alternative way, plant defense response against the pathogen can provide information indirectly for the virulence mechanism of *R. solani*. For this purpose, we treated Arabidopsis plants with defense-related phytohormones, SA, JA, and ET, 48 h prior to challenging the leaves with different *R. solani* isolates. The photographs taken at 0, 3, and 5 dpi are presented in Figure 3. In the case of the highly virulent isolates towards leaves, AG-1 IA (MAFF305230), AG-1 IC (MAFF243448), AG-2-1 II (MAFF305203), AG-2-3 (MAFF237259), AG-2-2 IIIB (MAFF726525), and AG-4 IIIA (MAFF305225), the symptoms were not altered in any phytohormone treatments. This means that the preactivation of the signaling cascades of these phytohormones did not provide an effective defense against these pathogenic isolates. Meanwhile, in the case of the non- or weak virulent isolates, SA slightly induced or enhanced chlorosis after the inoculation of the AG-2-2 IIIB (MAFF305244), AG-2-2 IV (MAFF241951), and AG-6 (MAFF305262). Similar results were observed after the treatment of ET in the AG-1 IA (MAFF305219 and MAFF305232), AG-2-2 IIIB (MAFF305244 and MAFF242301), AG-2-2 IV (MAFF241951), AG-2 BI (MAFF305228), AG-6 (MAFF305262), and AG-7 (MAFF305551). Although we did not measure the fungal biomass inside leaves, SA and ET seem to be working in favor of the pathogenicity of the fungus.

### 3.4. The Effects of Exogenously Applied SA, JA, and ET on the Resistance to Rhizoctonia solani in Arabidopsis Roots

**Figure 4:**
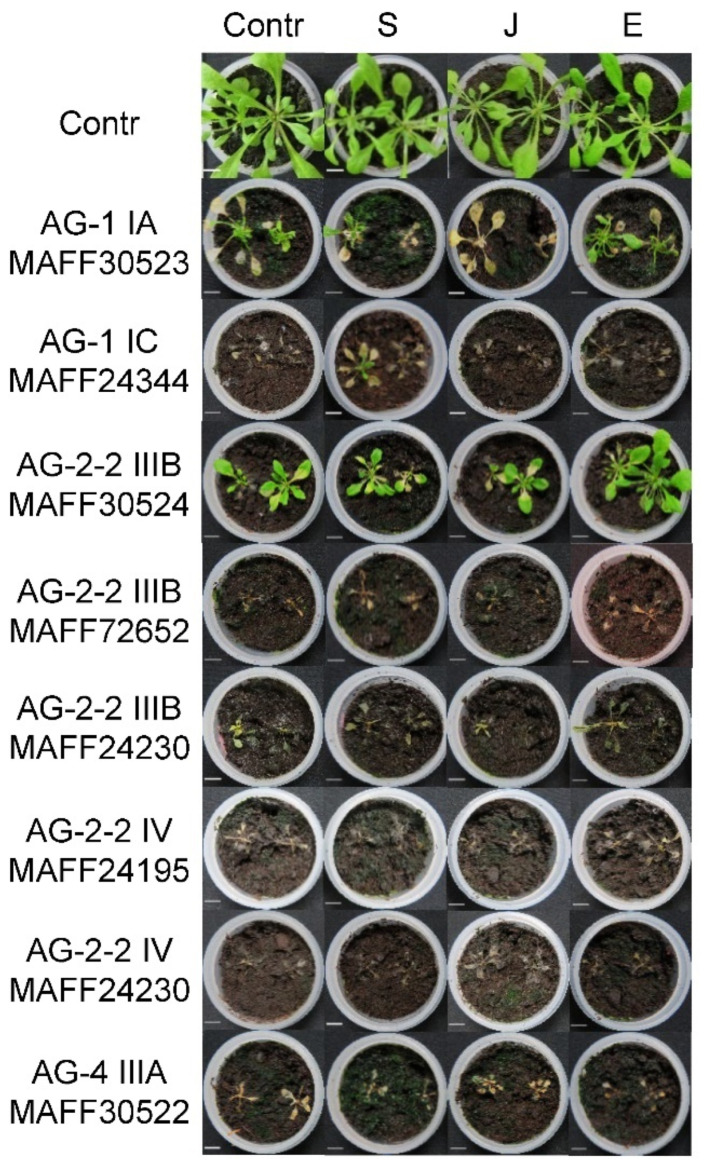
Effects of pretreatment of the defense-related phytohormones, salicylic acid (SA), jasmonic acid (JA), and ethylene (ET), on disease resistance of *Arabidopsis thaliana* plants to *Rhizoctonia solani* Japanese isolates inoculated into soil. Ten-day-old Arabidopsis seedlings grown on ½ MS medium were sprayed with SA, JA, or ET (1 mM each; final concentration of DMSO was 0.1%). Spraying of 0.1% DMSO was used as a control. After 48 h, the seedlings were transplanted into *R. solani*-inoculated soil. Soil inoculated with plain PDA plugs without fungus was used as a negative control. The *R. solani* isolates that exhibited strong virulence in this inoculation method as demonstrated in Figure 2 were tested. Photographs were taken at 14 days post-inoculation (dpi). Plain PDA plugs without fungus were used as a control. The assay was replicated three times.

We also tested the effects of SA, JA, and ET on the Arabidopsis resistance to the *R. solani* isolates in the soil inoculation assay. Ten-day-old Arabidopsis seedlings were treated with SA, JA, and ET 48 h before transplanting into the *R. solani*-inoculated soil. Photographs taken at 14 dpi are represented in Figure 4. In this experiment, we used the eight *R. solani* isolates that exhibited virulence in Figure 2. There were no obvious changes in symptoms and survival rate. This is similar to the results of the virulent isolates of *R. solani* for leaves as demonstrated in Figure 3.

## 4. Discussion

In this study, we evaluated the pathogenicity of three different AG-1 IA isolates, which were sampled from rice and sudangrass, on *A. thaliana*. We previously tested their virulence on *B. distachyon* and found that all of them were pathogenic [24]. These results suggest that they all have virulence mechanisms for monocotyledonous plants. Among them, only the AG-1 IA (MAFF305230) possesses pathogenicity to *A. thaliana*. It would have virulence mechanism(s) adapted to both monocots and dicots. This might make this isolate hypervirulent, as demonstrated on *B. distachyon*, where it had the highest pathogenicity ability compared with the other two isolates [24]. Maeda et al. reported that the Japanese isolate of *R. solani* AG-1 IA (MAFF243956) identified from rice fields is pathogenic to upper-ground parts of *A. thaliana* [34]. We confirmed this result and also found that it could induce necrotic symptoms in *A. thaliana* when it was inoculated to soil (Figure S1). MAFF305230 was isolated at Fukuoka in 1975, MAFF305219 was obtained at Fukuoka in 1966, and MAFF243956 came from Kagoshima in 1999. The comparative genomics approach will be able to reveal the molecular basis underlying these differences in host and tissue specificity.

The eight AG-2 isolates were tested in our study, and seven of them (all except AG-2 BI) were pathogenic to Arabidopsis. This is consistent with the previous consensus that AG-2 is relatively compatible with Brassicaceae [17]. The source of the AG-2 isolates used in this study is varied and it includes Poaceae, Brassicaceae, Amaryllidaceae, and Fabaceae. The AG-2-2 IIIB and AG-2-2 IV from broccoli and AG-2-2 IV from soybean exhibited pathogenicity to both upper- and underground tissues. The defense mechanisms of these plant species might be relatively similar; otherwise, these isolates acquired alternative pathogenicity genes for different host species in the past. The AG-2-1 II from six-rowed barley and AG-2-3 from rice have virulence to *A. thaliana* leaves but not roots. The AG-2-2 IIIB isolates from rice and welch onion have preferable virulence to roots. An AG-2-2 isolate obtained from sugar beet in Netherland was shown to be pathogenic to both Brassicaceae and Solanaceae [17]. These results suggest that the virulence mechanisms in the AG-2 group are not common and differ in each isolate. These isolates could be useful materials to study tissue-specific immunity mechanisms in plants and the virulence mechanism of *R. solani* for its breaking down.

The AG-4 IIIA used in this study was found to be pathogenic to both upper- and underground parts of *A. thaliana*. The source of this isolate is cauliflower; therefore, its virulence mechanism would be adaptable to the same family member *A. thaliana*. *R. solani* AG-4 HG-I and HG-II isolated from peanut and sugar beet, respectively, were demonstrated to be pathogenic to cauliflower, *A. thaliana*, and eggplant [17]. At this moment, it is unclear if the virulence mechanism in AG-4 isolates is varied or not. The AG-2 BI, AG-5, AG-6, and AG-7 used in this study are less or nonpathogenic to *A. thaliana.* This seems to be consistent with the fact that their sources are soil. Two *R. solani* AG-6 isolates in Japan were nonpathogenic to *A. thaliana* [17]. However, it does not exclude the possibility that these AG groups in nature contain virulent isolates to *A. thaliana* or exhibit virulence to other plant species. *R. solani* AG-5 GM-10 isolated from soybean in Japan showed moderate virulence to cauliflower and Arabidopsis [17]. Surveillance of virulence using at least several different isolates is needed to understand this point.

The pretreatment of the defense-related phytohormones SA, JA, and ET did not induce resistance in Arabidopsis to *R. solani* virulent isolates. This means that each defense mechanism mounted by these hormones is ineffective to cope with *R. solani*; otherwise, the pathogen can fully overcome them. This is inconsistent with the behavior of AG-1 IA on the SA-treated *B. distachyon* [24]. The disease resistance to *R. solani* was induced by the pretreatment of SA. Moreover, *B. distachyon* accessions Bd3-1 and Tek-3 are resistant to the *R. solani* isolate and activate SA-dependent gene expressions including BdWRKY38 and BdWRKY44. Furthermore, the resistance was shown to be dependent on BdWRKY38 but not BdWKRY44 [35]. These results suggested that the *R. solani* AG-1 IA would have a short biotrophic interaction at the early infection process on *B. distachyon*. This idea was also supported by the expression of the effector-like genes at the early infection stage up to 24 h prior to the formation of the necrotic lesions [36]. On the contrary, we also found that the exogenous application of BTH (acibenzolar-S-methyl) induced susceptibility of this pathogen in *B. distachyon*. Although BTH is thought to be a functional analog of SA, the transcriptome showed that the expression of more genes was altered in BTH treatment than in SA, and such differences would contribute to the BTH-induced susceptibility [37]. Considering the results observed in this study, *R. solani* was supposed not to undergo a biotrophic stage on Arabidopsis. This is according to the results that the susceptibility to the *R. solani* AG-2-1 and the resistance to the nonhost *R. solani* AG-8 were not changed in Arabidopsis *NahG* plants and *npr1-5* mutant [21]. In this study, we evaluated the disease severity with visible symptoms. Therefore, the pretreatment of the phytohormones is still possible to confer slight resistance that can be detected by in planta fungal biomass or microscopic observations supported by statistical analyses.

In our study, the exogenous application of ET and SA slightly enhanced the susceptibility of Arabidopsis leaves to most of the *R. solani* isolates with non- or weak virulence. Consistently, the exogenous application of ET was found to enhance disease development by *Botrytis cinerea* in various plants [38]. Because ET and SA are known to be involved in senescence, such activities may accelerate symptom development [39,40]. Because the phytohormone treatments did not induce apparent resistance or susceptibility, it suggests that the defense layers in Arabidopsis against *R. solani* are somehow more complicated. The resistance level of Arabidopsis to the nonadapted *R. solani* AG-8 isolate was found to be reduced in *coi1 ein2 pen2* mutants. Therefore, this nonhost resistance would be composed of multiple defense layers including JA and ET [20]. The AG-1 IA (MAFF305230) would possess a virulence mechanism for monocotyledonous plants as well as deploy weapons to suppress the defense responses induced by JA and ET and *PEN2*-dependent immunity in Arabidopsis. Because the AG-1 IA isolates (MAFF305219 and MAFF305232) are pathogenic to rice and sudangrass, respectively, but not to Arabidopsis, they may lack at least one of such virulence pieces of machinery. Confirmation using the mutants in multiple genes is needed to understand the virulence mechanism of *R. solani* in dicotyledonous plants in the future.

## Figures and Tables

**Table 1:** *Rhizoctonia solani* isolates used in this study and their pathogenicity to upper- and underground tissues of *Arabidopsis thaliana* Col-0.

AGs	MAFF Number ^1^	Name	Growth (Days) ^2^	Source	Infection Severity ^3^	
					Leaves	Roots
AG-1 IA	305230	C-325	3	Rice	+++	++
AG-1 IA	305219	C-54	6	Rice	−	−
AG-1 IA	305232	C-501	3	Sudangrass	−	−
AG-1 IC	243448	RD1	3	Carrot	+++	+++
AG-2-1 II	305203	6	7	Six-rowed barley	++	−
AG-2-2 IIIB	305244	C-329	7	Rice	−	++
AG-2-2 IIIB	726525	RS-B	3	Broccoli	+++	+++
AG-2-2 IIIB	242301	WLS81	4	Welch onion	−	+++
AG-2-2 IV	241951	SBF1	4	Broccoli	++	+++
AG-2-2 IV	242303	SD1	4	Soybean	++	+++
AG-2-3	237259	H5-210	7	Wheat	++	−
AG-2 BI	305228	SH-1-2	7	Soil	−	−
AG-3 IV	305250	C564	14	Potato	−	−
AG-4 IIIA	305225	BO-3	3	Cauliflower	+++	+++
AG-5	305256	SH-30	7	Soil	+	−
AG-6	305262	UB-7-1-A	4	Soil	−	−
AG-7	305551	1529	3	Radish field soil	+	−

^1^ MAFF numbers are descriptors for the microorganism genetic resources assigned by NARO (National Agriculture and Food Research Organization) Genebank in Japan. ^2^ Days taken by the fungus to fill the 90 mm petri plate. ^3^ Infection severity visually assessed, (+++) severe symptoms, (++) moderate symptoms, (+) weak symptoms, (−) no symptoms.

## Supplementary Materials

The following supporting information can be downloaded at: https://www.mdpi.com/article/10.3390/life12010076/s1, Figure S1: Infectivity of *Rhizoctonia solani* Japanese isolate AG-1 IA (MAFF243956) in upper- and underground tissues of *Arabidopsis thaliana*.
