# Supplementary material for: Surveillance of Pathogenicity of Rhizoctonia solani Japanese Isolates with Varied Anastomosis Groups and Subgroups on Arabidopsis thaliana
Source: Life (Basel). 2022 Jan 6;12(1):76. doi: 10.3390/life12010076 (PMC8781544; doi:10.3390/life12010076)
Supplement: Supplementary file 1 [file life-12-00076-s001.zip › life-1540108-supplementary.pdf]

life

Supplementary

Surveillance of Pathogenicity of *Rhizoctonia solani* Japanese Isolates with Varied Anastomosis Groups and Subgroups on *Arabidopsis thaliana*

Mai Mohsen Ahmed Abdelghany [1,2], Maria Kurikawa [3], Megumi Watanabe [1], Hidenori Matsui [1,3], Mikihiro Yamamoto [1,3], Yuki Ichinose [1,3], Kazuhiro Toyoda [1,3], Yusuke Kouzai [1,†] and Yoshiteru Noutoshi [1,3,*]

1 Graduate School of Environmental and Life Science, Okayama University, Okayama 700-8530, Japan; pazi0ct5@s.okayama-u.ac.jp (M.M.A.A.); po7l1am9@okayama-u.ac.jp (M.W.); myama@okayama-u.ac.jp (M.Y.); hmatsui@okayama-u.ac.jp (H.M.); yuki@okayama-u.ac.jp (Y.I.); pisatin@okayama-u.ac.jp (K.T.); yusuke.k@affrc.go.jp (Y.K.)
2 National Institute of Oceanography and Fisheries (NIOF), Alexandria 21556, Egypt
3 Department of Agriculture, Okayama University, Okayama 700-8530, Japan; p3io9bg4@s.okayama-u.ac.jp
* Correspondence: noutoshi@okayama-u.ac.jp
† Present address: Crop Stress Management Group, Division of Plant Molecular Regulation Research, Institute of Agrobiological Sciences, National Agricultural and Food Research Organization (NARO), Tsukuba, Ibaraki 305-8518, Japan.

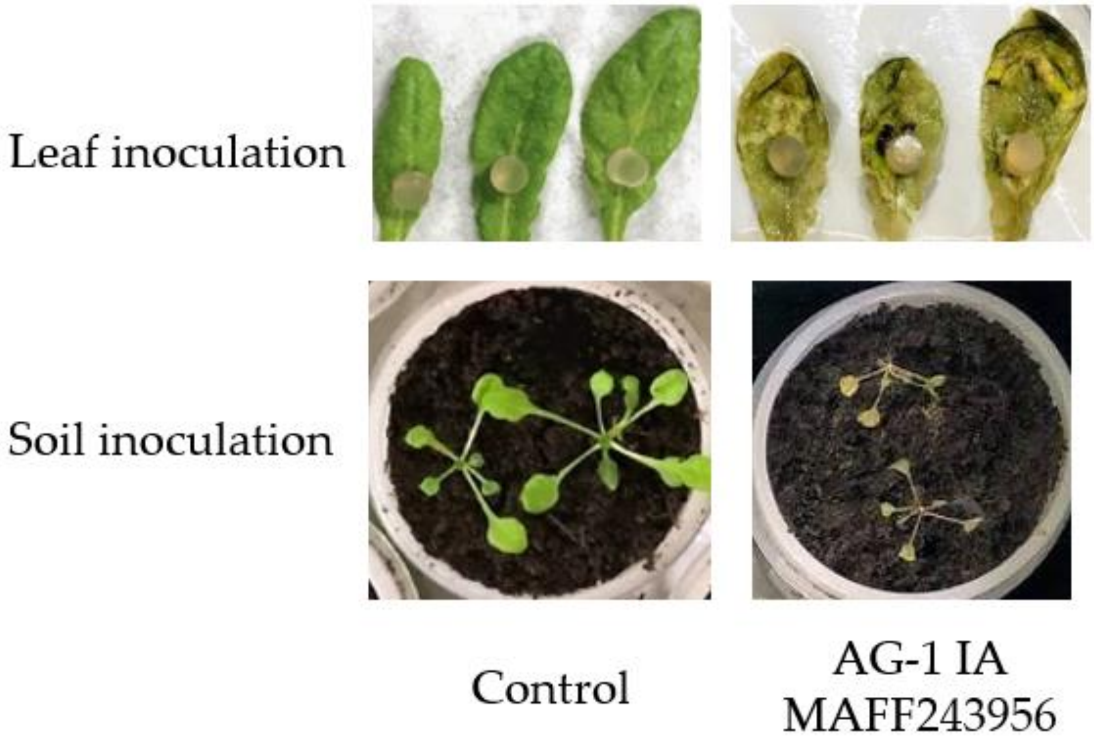

Figure S1. Infectivity of *Rhizoctonia solani* Japanese isolate AG-1 IA (MAFF243956) in upper and underground tissues of *Arabidopsis thaliana*. Leaves were inoculated with 6-mm mycelial PDA plugs of the *R. solani* and incubated in a humid condition at 23 °C for 5 days. Photographs were taken at 7 days post-inoculation (dpi). Plain PDA plugs without fungus were used as a control. For soil inoculation, mycelial plugs were inoculated to soil and incubated for 6 days. Then, 2-week-old Arabidopsis seedlings grown on MS agar plate medium were transplanted. Photographs were taken at 14 dpi. Plain PDA plugs without fungus were used in the control treatment. The assays were performed three times with three biological replicates.
